# The Stomach-Tube—Its Origin, Use and Abuse

**Published:** 1904-11

**Authors:** L. Amster

**Affiliations:** Atlanta, Ga.


ATLANTA

### Journal-Record of Medicine.

Successor to Atlanta Medical and Surgical Journal, Established 1855, and Southern Medical Record, Established 1870.

OWNED BY THE ATLANTA MEDICAL JOURNAL CO.

Published Monthly.

VOL. VI. NOVEMBER, 1904. No. 8.

BERNARD WOLFF, M.D., EDITOR, Nos. 319-20 Prudential.

M. B. HUTCHINS, M.D., BUSINESS MANAGER, 1007-1008 Century Bldg.

J. N. LECONTE, M D., ASSOCIATE EDITOR.

### ORIGINAL COMMUNICATIONS.

### THE STOMACH-TUBE—ITS ORIGIN, USE AND ABUSE.

BY L. AMSTER, M.D.,

ATLANTA, GA.

Great things that are the means of accomplishing much good are often the cause of much mischief through the abuse to which they are subjected.

Prompted by the knowledge of how indiscriminately the stomach-tube is being used, how much suffering and harm is being done daily by this great diagnostic and therapeutic agent, I can not help grasping this opportunity to record my veto against a procedure which is bringing the honest and legitimate practice of diseases of the gastro-intestinal tract into disrepute, and turning the attention of patients and sufferers to nostrums aud quackery.

It would be folly to expect of the general practitioner that he be master of the whole subject of every specialty, but there are certain parts and phases with which he ought to be familiar. In gastro-intestinal work, it is the intelligent use of the stomach-tube. It is the foundation and corner-stone for the diagnosis and treatment of diseases of the stomach and intestines.

Considering its vast importance, it will be of interest to trace its origin.

The English arrogate to themselves the invention of the stomach-tube. It is generally assumed that two English surgeons, Jukes and Bush, were the first who used it. With pardonable pride, we are able to refute this claim, by pointing to positive evidence that it was an American physician who first used the stomach-tube for therapeutic purposes.

The claim of the English is based upon an article published by Jukes in the *London Medical Repository* of 1822, an abstract of which is found in the *American Medical Recorder* of 1823.

Dr. Physick, an American physician, published a paper in October, 1812, in the *Eclectic Repertory* Vol. III, page 111, under the title of "Account of a New Mode of Extracting Poisonous Substances from the Stomach. By Philip S. Physick, M.D., Professor of Surgery in the University of Pennsylvania." The article appeared about ten years before Jukes' article.

Dr. Physick, in his article, describes a case of two children, twins, who had swallowed laudanum and were in an extremely critical condition. The children were unable to swallow an emetic. As under the circumstances no time was to be lost, he determined to inject an emetic into their stomachs. For this purpose, a large, flexible catheter was passed through the mouth, down the esophagus into the stomach, and through this one drachm of ipecacuanha, mixed with water, was quickly passed by means of a common pewter syringe. As no emesis was produced, he drew the fluid contents of the stomach and immediately injected warm water, which was again withdrawn. This procedure was repeated a few times. Then he injected some spirits and water, mixed with a little vinegar. The children reacted, and their lives were saved. He goes on to say that the idea of washing out the stomach with a syringe and tube, in cases of poisoning, occurred to him twelve years before and that he had constantly for many years recommended the method in his lectures.

That the credit of this invention is due Dr. Physick is also shown by Matthews in the *Medical Recorder* Volume X, 1826, page 325, in an article entitled "Description of an Improved Instrument for Extracting Poison from the Stomach; with some Statements Tending to Establish the Validity of Dr. Physick's Title to the Credit of having Invented the Stomach-tube."

There are testimonials by three American physicians who attended Dr. Physick's lectures: Isaac Cleaver testifies that he heard Dr. Physick recommend the use of the stomach-tube in 1802-3-4. John D. Thomas attended Dr. Physick's lectures in the 1805-6-7, and that in these lectures Dr. Physick exhibited a tube, coated with elastic gum, brought by Dr. Dorsey from Paris and made there by Dr. Physick's order. Benjamin L. Jerry remembered distinctly that in the winter of 1808-9, Dr. Physick informed him that he had an elastic tube brought from Europe by Dr. Dorsey for the purpose of washing out the stomach.

In the year 1803, Dr. Dorsey was in Paris and had made there, by Dr. Physick's order, a tube constituted of the same material as the French catheter. The use of this tube was unknown in Paris and the makers were curious to know what the intention was of so large a catheter.

Dr. Alexander Munro, Jr., an English physician, in his inaugural thesis, published in 1799, suggests the use of the tube in cases of poisoning for the extraction of poison from the stomach, and for the introduction of food in cases of dysphagia, and for extracting gases and food from the stomach in cases of gastric fermentation in cattle. He does not point out, however, that any practical application of the tube was made in cases of poisoning.

Dr. Physick admits that Dr. Munro had made his plan public before his own publication, but he never came across Dr. Munro's publication until after he himself had used the stomach-tube for years. No credit is due to Dukes and Bush, who are generally considered the originators of the stomach-tube. There can be no doubt that the invention and practical application was due to the American.

Apart from previous occasional sporadic efforts to introduce the stomach-tube generally into practice, Kussmaul was the first one who, in the year 1867, demonstrated its high therapeutic value in a number of cases of gastric dilatation. Gradually it found its way into the armamentarium of the physician, until Leube, in 1871, first recommended the stomach-tube for diagnostic purposes.

Not until 1880 did it become firmly established as a diagnostic and therapeutic agent—thereby laying the foundation for the better understanding of gastro-intestinal diseases and their treatment.

For diagnostic purposes the stomach-tube serves:

1. To test the permeability of the esophagus (stricture, foreign bodies).

2. To diagnose certain conditions of the esophagus, as dilatation and diverticles.

3. To define the position and size of the stomach.

4. To establish the motor-power of the stomach.

5. To withdraw the stomach contents for the purpose of chemical examination.

According to Boas, we may say, in a general way, that the use of the stomach-tube for diagnostic purposes is indicated in those cases in which it is difficult or impossible to make a positive diagnosis.

Riegel, on the contrary, recommends the use of it in any case, if only for the confirmation of the diagnosis, unless absolutely contraindicated by certain conditions of the patient; as the physician often is certain of his diagnosis, he still may find himself mistaken after eliciting other diagnostic points by the use of the tube.

Boas briefly sums up the contraindications; wherever its use is fraught with danger to the patient, or where the symptoms of the case point to a positive diagnosis.

To enumerate in detail the contraindications to the explorative use of the stomach-tube:

1. In all constitutional and local conditions which could be aggravated, or life-endangered, by the irritation and exertion produced by the tube.

Among these are:

(a) Heart disease, with defective compensation, angina pectoris, myocarditis, fatty heart in an advanced stage.

(b) Aneurism of the larger arteries.

(c) Recent hemorrhage (brain, lung, renal, vesical, gastro-intestinal, uterine hemorrhages and hemorrhagic infractions).

(d) Advanced pulmonary tuberculosis.

(e) Advanced pulmonary emphysema, with bronchitis.

(f) Complete and incomplete apoplexy, anemia of the brain and epilepsy.

(g) Pregnancy.

(h) Presence of continued and remittent fever.

2. Stomach and intestinal affections that can be diagnosed without the aid of the tube; these are:

(a) Ulcer, with recent hematemesis and black stools, due to blood.

(b) Palpable carcinoma of the stomach, with coffee-ground vomit and the other classical symptoms of carcinoma.

(c) Various neuroses of the stomach, which can be easily recognized by their symptoms.

(d) Acute febrile stomach and intestinal affections.

(e) Easily bleeding gastric mucosa.

(f) Secondary gastric affections due to some evident primary disease (phthisis, nephritis, diabetes, gall-stones, etc.)

3. During menstruation, according to investigations of Hans Elsner, analyses of the stomach contents at menstrual time show deviation from the findings at intermenstrual times, but the motor function remains the same.

Of course there may be occasions where the physician may find the use of the stomach-tube indispensable, in spite of existing contraindications. These, however, are exceptions which must be left to the sound judgment of the physician. A case of poisoning, *e. g.*, where human life is in immediate danger, must naturally do away with other considerations. For therapeutic purposes the stomach-tube is employed:

(a) For lavage in stagnation of the esophagus (dilatation or diverticle).

(b) For the dilation of stricture of the esophagus and the instillation of oil.

(c) For the removal of poisonous substances ingested.

(d) For lavage in dilatation of the stomach.

(e) Lavage in pylorospasms and stricture of the pylorus, which are inoperable, or where the patient refuses the radical operation.

(f) Lavage in gastritis, with an abundance of mucous secretion.

(g) Lavage in gastric neurosis for its psychic effect.

(h) Lavage in gastrosuccorhea, or Reichman's disease.

(i) Boas observed good results from lavage in pregnancy in carefully selected cases, where other treatments failed.

(j) Lavage in insane patients, or other patients refusing to swallow food.

(k) Lavage in diseases of the esophagus, where food can not naturally reach the stomach.

(l) Louis Fisher, of New York, demonstrated to me the great utility of lavage in cases of intubation after diphtheria and other stenoses of the larynx.

When I first announced to my friends and patients and to some of my medical brethren that I had decided to give up general practice and devote myself to gastro-intestinal diseases, I was asked such questions as these: Are you going into the stomach-washing business? Are you going to run a stomach laundry? Or, are you going to have a stomach lavatory? I thought these were innocent pleasantries, but it was soon brought home to me that these remarks contained a deep meaning with a moral.

Most of my patients, upon entering my office, say that they would like to be relieved of their ailments, upon the condition of my not washing out their stomachs. There has hardly been any exception; almost all, who have been treated before, have had lavage. I found many were not suffering from stomach trouble at all, but that they had either some intestinal ailment, gall-bladder trouble, enteroptosis or nephroptosis. Some did have stomach trouble, where lavage could do no good, and in one case of gastric ulcer, the patient had had daily lavage, in spite of small hemorrhages and such discomfort and pain that she had to go to bed after each ordeal.

No wonder people are afraid of such indiscriminate practice. They are imbued with the idea that the scientific treatment of diseases of the stomach is synonymous with stomach-washing, and it is not surprising if they turn to the more pleasant and convenient methods of quacks and agreeable-tasting nostrums.

It is reported that an institution exists in the South where the treatment of all cases, regardless of their nature, consists in the eating of some kind of magic hot biscuits baked in oil, and stomach washing. Washing externally, internally and eternally, without rhyme or reason or mercy. Evidently such an institution takes the adage that "cleanliness is next to godliness" a trifle too literally.

Gentlemen, such glaring abuse of this valuable method of treatment will never do. It may, for a time, tend to increase one's revenue, but people are sure to find out that they are being imposed upon, and discredit to our honored profession is the result of such maltreatment.

In properly selecting cases, where the stomach tube is indicated, it will surely fulfill its mission and will gain for the physician the gratitude of the patient. Just as soon as we abuse its employment the reverse is bound to follow.

During the time of my attendance at the policlinics of such eminent men as Boas, Ewald and Conheim, with their large and varied material, lavage was rarely used. The Boas School is very conservative as to the use of the stomach-tube for therapeutic purposes. For Boas, there is only one cardinal indication for lavage and that is "Stagnation of stomach contents." Even then he thinks simple expression of stagnating stomach contents, without lavage, is sufficient in the majority of cases. In gastrosuccorrhea, also, simple removal of the accumulated gastric juice, is sufficient to accomplish the desired result.

In gastritis, with abundant mucous, he prefers to administer solving agents.

Any method of treatment applied indiscriminately degrades the physician to the level of the charlatan. Our ambition must be to apply reason and science in diagnosing our cases:

"*Qui bene diagnoscit, bene curat.*"

Furthermore, every physician using the stomach-tube should remember that the introduction of it may be very simple, nevertheless, to the patient, it is by no means a pleasurable procedure. We have to apply quite frequently the tube for diagnostic purposes, but we must not inflict on our patients more discomfort than is conducive with the clearest indication.

Lavage often produces pain, shock and exhaustion and should not be persisted in in such cases. I fear that dilatation is often produced in cases of atony of the stomach, where large quantities of fluid are thrown into a weakened organ for any length of time.

I shall, therefore, conclude with the admonition: Use the stomach-tube when you must, but be careful that its use does not degenerate into abuse.

References: Friedenwald, Boas, Riegel.

